# A High-Affinity ^64^Cu-Labeled Ligand for PET Imaging of Hepsin: Design, Synthesis, and Characterization

**DOI:** 10.3390/ph15091109

**Published:** 2022-09-05

**Authors:** Ji-Hun Park, Xuran Zhang, Hyunsoo Ha, Jung Young Kim, Joon Young Choi, Kyung-Han Lee, Youngjoo Byun, Yearn Seong Choe

**Affiliations:** 1 Department of Nuclear Medicine, Samsung Medical Center, Sungkyunkwan University School of Medicine, Seoul 06351, Korea; 2 College of Pharmacy, Korea University, Sejong 30019, Korea; 3 Division of Applied RI, Korea Institute of Radiological and Medical Sciences, Seoul 01812, Korea; 4 Department of Health Sciences and Technology, SAIHST, Sungkyunkwan University, Seoul 06355, Korea

**Keywords:** hepsin, prostate cancer, ^64^Cu-DOTA-conjugated ligand, Leu–Arg dipeptide derivatives, diastereomers, PET

## Abstract

Hepsin, a cell surface serine protease, is a potential biomarker for the detection of prostate cancer due to its high expression in prostate cancer but not in normal prostate. This study aimed to develop a radioligand for positron emission tomography (PET) imaging of hepsin. Six leucine–arginine (Leu–Arg) dipeptide derivatives (two diastereomers for each of three ligands) were synthesized and evaluated for their binding affinities and selectivity for hepsin. Based on the binding assay, a ^nat^Cu-1,4,7,10-tetraazacyclododecane-*N*,*N*′,*N*″,*N*‴-tetraacetic acid (DOTA)-conjugated ligand (**3B**) was selected for the development of a PET radioligand. [^64^Cu]**3B** was synthesized by labeling the DOTA-conjugated compound **11B** with [^64^Cu]CuCl_2_ at 80 ^°^C for 20 min. The radioligand was evaluated for prostate cancer cell binding and PET imaging in a prostate tumor mouse model. The results demonstrated that [^64^Cu]**3B** exhibited high binding to LNCaP cells, intermediate binding to 22Rv1 cells, and low binding to PC3 cells. PET studies of [^64^Cu]**3B** in mice, implanted with 22Rv1 and PC3 cells on each flank, revealed that the radioligand uptake was high and persistent in the 22Rv1 tumors over time, whereas it was low in PC3 tumors. The results of this study suggest that [^64^Cu]**3B** is a promising PET radioligand for hepsin imaging.

## 1. Introduction

Prostate cancer is the second most common cancer following lung cancer and represents the sixth leading cause of cancer-related death among men worldwide [1]. In the United States, prostate cancer is the most common cancer and the second leading cause of cancer-related death among men [2]. Prostate cancer that is still confined within the organ can be treated by surgery and radiation therapy [3]. However, metastasis of this cancer into other organs may increase the mortality rate [4]. Therefore, early detection of prostate cancer is essential for an improved prognosis after therapy. The serum prostate-specific antigen (PSA) test is widely used for the early screening of prostate cancer [5]. However, the serum PSA levels are not specific for prostate cancer, because PSA is expressed not only in prostate cancer but also in the normal prostate. This makes discrimination between normal prostate or benign prostatic hyperplasia (BPH) and prostate cancer challenging [6]. Therefore, specific molecular biomarkers for prostate cancer are required for early and accurate diagnosis of the disease. 

Hepsin is a type II transmembrane serine protease composed of 413 amino acids. Tissue microarray-based expression profiling demonstrated that hepsin expression was upregulated in prostate cancer specimens, but low or no hepsin expression was detected in normal prostate and BPH specimens [7,8,9,10,11]. Additionally, increased expression of hepsin has been observed in metastatic tumors compared to primary tumors [7,11]. Moreover, the crystal structure of soluble human hepsin exhibits that two domains are located extracellularly and that the larger domain contains the active site cleft for the binding of hepsin inhibitors [12]. These characteristics render hepsin a potential biomarker for the diagnosis of prostate cancer. 

Over the past few decades, ligands have been specifically developed for molecular imaging of hepsin. Hepsin-binding peptides with high affinity (190 nM) and selectivity were identified using phage display [13]. When analyzed by fluorescence-activated cell sorting, multiple FITC-peptides conjugated to fluorescent nanoparticles (Cy5.5) (IPL-NP) exhibit a markedly increased signal in hepsin-expressing prostate cancer cells compared with the monomeric FITC-peptide. Using fluorescence-mediated tomography, small tumors were detected in mice injected with IPL-NP and its accumulation was 2.8-fold higher in hepsin-positive LNCaP tumors than in hepsin-negative PC3 tumors. In another study, a heterobivalent ligand, targeting both hepsin and prostate-specific membrane antigen (PSMA), was synthesized. Its SulfoCy7-conjugated version was evaluated in vitro and in vivo [14]. The ligand displayed inhibitory activities for hepsin (IC_50_ = 2.8 μM) and PSMA (IC_50_ = 28 nM). The near-infrared (NIR) images of mice showed significantly stronger signals in PC3/ML–PSMA–hepsin tumors than in low hepsin expressing PC3/ML tumors [14]. Moreover, stronger signals were observed in PC3/ML–PSMA–hepsin tumors compared to those in PC3/ML–PSMA tumors. More recently, BODIPY- and SulfoCy7-conjugated Leu–Arg dipeptide derivatives were developed for the NIR imaging of hepsin [15]. Despite their high binding affinities for hepsin (*K*_i_ = 21 and 22 nM), the ligands were not evaluated for NIR imaging. As such, small molecule hepsin inhibitors have been developed for optical imaging. However, none have been applied to PET imaging so far. 

**Figure 1:**
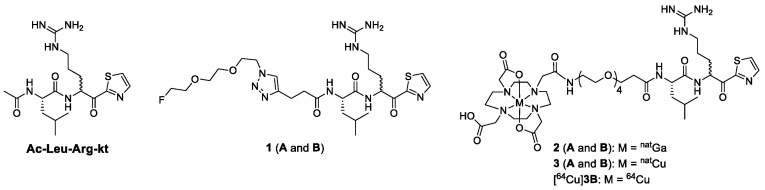
Structures of Ac–Leu–Arg–kt, ligands **1**–**3**, and [^64^Cu]**3B**.

In the present study, we developed a PET radioligand that was derived from a Leu–Arg dipeptide-based hepsin inhibitor [15]. We synthesized six Leu–Arg dipeptide derivatives (two diastereomers for each of three ligands) (Figure 1): fluorine-substituted ligands (**1A** and **1B**), ^nat^Ga-DOTA-conjugated ligands (**2A** and **2B**), and ^nat^Cu-DOTA-conjugated ligands **(3A** and **3B**). Based on our findings with respect to the binding affinity and selectivity for hepsin, **3B** was selected for radiolabeling. The ^64^Cu-DOTA-conjugated ligand, [^64^Cu]**3B** (Figure 1) was synthesized and characterized for PET imaging of hepsin.

## 2. Results

### 2.1. Chemical Synthesis

**Scheme 1:**
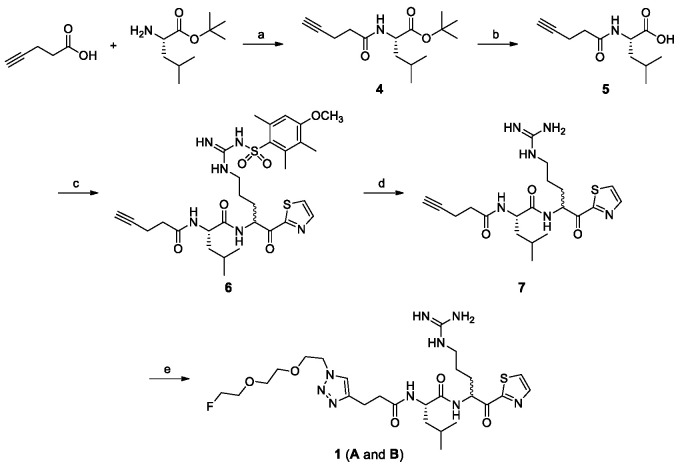
Reagents and conditions: (**a**) HOBt, EDC·HCl, CH_2_Cl_2_, room temperature, 3 h; (**b**) TFA, CH_2_Cl_2_, room temperature, 2 h; (**c**) HOBt, EDC·HCl, CH_2_Cl_2_, NH_2_–Arg(Mtr)–kt, Et_3_N, room temperature, 3 h; (**d**) TFA/thioanisole/water (95/2.5/2.5), room temperature, 3 h; (**e**) 1-azido-2-(2-(2-fluoroethoxy)ethoxy)ethane, CuSO_4_·5H_2_O, sodium ascorbate, EtOH/water, room temperature, 3 h.

Non-radioactive ligands **1**–**3** were designed and synthesized for the development of a PET imaging agent for hepsin (Figure 1). Mini-polyethylene glycol linkers were introduced to the Leu–Arg dipeptide derivatives in order to increase the hydrophilicity and improve the in vivo properties of the (radio)ligands [15,16,17,18,19,20]. Ligand **1** was synthesized via a click reaction between the terminal alkyne compound **7** and 1-azido-2-(2-(2-fluoroethoxy)ethoxy)ethane (Scheme 1). Compound **7** was synthesized from 4-pentynoic acid and l-leucine *tert*-butyl ester in four steps; removal of the *tert*-butyl group of compound **4** gave compound **5**, which was then reacted with *N*-(*N*-(4-amino-5-oxo-5-(thiazol-2-yl)pentyl)carbamimidoyl)-4-methoxy-2,3,6-trimethylbenzenesulfonamide (NH_2_–Arg(Mtr)–kt) [21] to yield compound **6**, and the subsequent deprotection of the Mtr group of **6** yielded **7**. Compound **7** was obtained as a 1:1.7 ratio of diastereomers based on the HPLC analysis, although the NH_2_–Arg(Mtr)–kt used for synthesis of **6** was prepared from *tert*-butoxycarbonyl (BOC)–NH–l-Arg(Mtr)–OH in three steps [21]. Ligand **1** was also obtained as a 1:1.7 ratio of diastereomers (**A** and **B**), and the yield was quantitative based on compound **7** when analyzed by HPLC. The ligand was purified by HPLC to yield **1A** and **1B**.

**Scheme 2:**
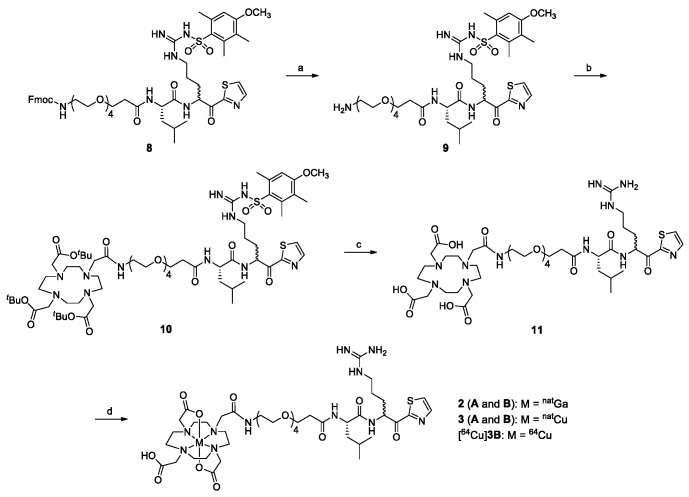
Reagents and conditions: (**a**) piperidine, CH_2_Cl_2_, room temperature, 2 h; (**b**) DOTA-tris(*tert*-butyl)ester NHS ester∙HPF_6_, Et_3_N, CH_2_Cl_2_, room temperature, 2 h; (**c**) TFA/thioanisole/water (95/2.5/2.5), room temperature, 6 h; (**d**) **2**: Ga(NO_3_)_3_·xH_2_O, sodium acetate buffer (pH 6), room temperature, 16 h; **3**: CuCl_2_·2H_2_O, water, room temperature, 16 h; [^64^Cu]**3B**: [^64^Cu]CuCl_2_, sodium acetate buffer (pH 6), 80 °C, 20 min.

Ligands **2** and **3** were obtained by reacting DOTA-conjugated compound **11** with Ga(NO_3_)_3_·xH_2_O and CuCl_2_·2H_2_O, respectively (Scheme 2). Compound **11** was prepared from compound **8** in three steps. Compound **8** was synthesized as reported previously [15,21]. Removal of the Fmoc group of compound **8** resulted in **9**. The reaction of **9** with DOTA-tris(*tert*-butyl)ester NHS ester, followed by deprotection of both *tert*-butyl and Mtr groups, yielded compound **11**. The arginine moiety of **8** is reported to exist as diastereomers [15], although the NH_2_–Arg(Mtr)–kt used for synthesis of **8** was prepared from BOC–NH–l-Arg(Mtr)–OH in three steps [21]. Ligands **2** and **3** were obtained as a 1:1 ratio of diastereomers (**A** and **B**) and the yield was quantitative based on compound **11** by HPLC analysis. Each ligand was purified using HPLC to yield A and B ligands. Compound **11** was further purified as **11A** and **11B** using HPLC for ^64^Cu-labeling. 

The two diastereomers, A and B isomers, were not identified. Therefore, we designated the peaks eluted at the earlier and later retention times on HPLC as A and B isomers, respectively. 

### 2.2. Radiochemical Synthesis

The radioligand was synthesized by labeling the DOTA-conjugated compound **11B** with [^64^Cu]CuCl_2_ at 80 °C for 20 min (Scheme 2). The subsequent HPLC purification gave [^64^Cu]**3B** with an overall 28–43% decay-corrected radiochemical yield, >98% radiochemical purity, and 10–12 GBq/μmol molar activity. The total synthesis time, including HPLC purification followed by formulation, was 90–100 min. 

### 2.3. In Vitro Binding Assay

The binding affinities of all six ligands for hepsin were measured (Figure S1). The B isomers exhibited higher binding affinities for hepsin compared to A isomers (Table 1). Binding affinities of the ligands for matriptase, a well-characterized serine protease [22], were also measured to investigate the selectivity of the ligands for hepsin. Similar binding patterns of the ligands were observed; B isomers showed higher binding affinities for matriptase as compared to A isomers. The majority of the ligands displayed significantly higher binding affinities and selectivity for hepsin compared with the Ac–Leu–Arg–kt (Figure 1), which is a known hepsin inhibitor [23]. The *K*_i_ value and hepsin selectivity (ratio of *K*_i_ values for matriptase and hepsin) of Ac–Leu–Arg–kt measured in this study were 7.8 ± 2.8 nM and 7.2, while the previous reported values were 22.4 ± 0.50 nM and 14.9, respectively [23]. Fluorine-substituted ligands **1A** and **1B** exhibited the highest binding affinities (*K*_i_ = 0.9 ± 0.1 nM and 0.5 ± 0.1 nM, respectively), whereas ^nat^Ga- and ^nat^Cu-DOTA-conjugated ligands showed binding affinities comparable to each other (**2A** and **3A**: *K*_i_ = 14.3 and 15.0 nM; **2B** and **3B**: *K*_i_ = 5.7 and 5.1 nM, respectively). Hepsin selectivity over matriptase was also higher for **1** (60.6 for A isomer and 40 for B isomer) than that for **2** (11.6 and 12 for A and B isomers, respectively) and **3** (16.6 and 23.5 for A and B isomers, respectively) (Table 1). Ligand **3B** was selected for radiolabeling and for further evaluation, because [^64^Cu]**3B** had the potential to be developed as a theranostic pair with the ligand labeled with a therapeutic radionuclide, such as ^67^Cu.

### 2.4. In Vitro Serum Stability 

**Figure 2:**
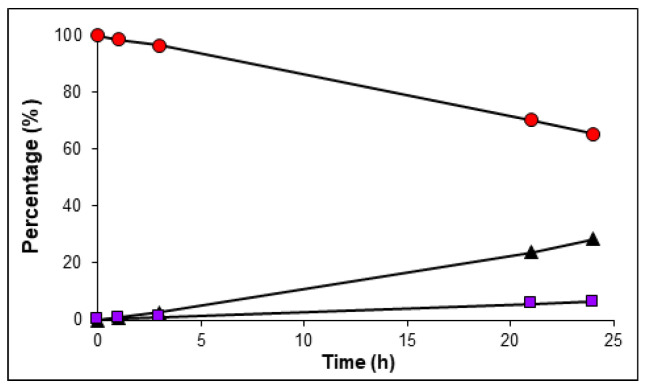
In vitro stability of [^64^Cu]**3B** in FBS at 37 °C. The radioligand was slowly epimerized to [^64^Cu]**3A** over time. [^64^Cu]**3B** (○); [^64^Cu]**3A** (△); an unidentified radioactive impurity (□). The unidentified radioactive peak at 4.5 min was 6.3% at 24 h.

An incubation mixture of the radioligand in fetal bovine serum (FBS) was analyzed using HPLC at the indicated time points (Figure S2). An unidentified radioactive peak appeared at 4.5 min and increased slightly over time: 0.2% at 0 h, 0.7% at 1 h, 1.0% at 3 h, 5.8% at 21 h, and 6.3% at 24 h (Figure 2). During this period, the [^64^Cu]**3B** was slowly epimerized to the [^64^Cu]**3A**; [^64^Cu]**3B** (99.8% at 0 h, 98.5% at 1 h, 96.3% at 3 h, 70.3% at 21 h, and 65.3% at 24 h) and [^64^Cu]**3A** (0% at 0 h, 0.8% at 1 h, 2.7% at 3 h, 23.9% at 21 h, and 28.4% at 24 h) (Figure 2). Although the [^64^Cu]**3B** was gradually epimerized to the [^64^Cu]**3A** over time, both **3A** and **3B** exhibited high binding affinities and selectivity for hepsin (Table 1). Moreover, the unidentified polar radioactive peak at 4.5 min was less than 6.3% at 24 h. Therefore, the [^64^Cu]**3B** was further studied in vitro and in vivo.

### 2.5. Cell Binding

**Figure 3:**
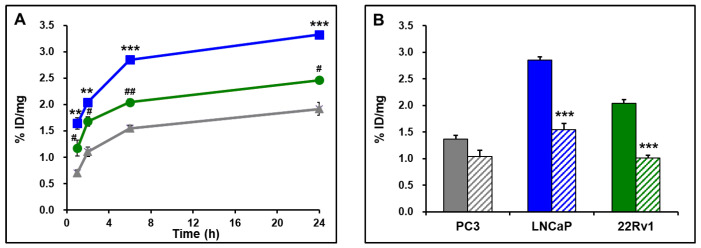
(**A**) Binding of [^64^Cu]**3B** to prostate cancer cells (PC3 (△), 22Rv1 (○), and LNCaP (□)) at 37 °C for 1, 2, 6, and 24 h. Data are expressed as mean ± SEM. ** *p* < 0.01 and *** *p* < 0.001 vs. PC3. ^#^ *P* < 0.05 and ^##^ *p* < 0.01 vs. PC3. (**B**) Blocking of the cell binding in the presence of **3B** (20 μM) at 37 °C for 6 h. Control: PC3 (grey), LNCaP (blue), and 22Rv1 (green) cells; block: PC3 (grey stripe), LNCaP (blue stripe), and 22Rv1 (green stripe). *** p < 0.001 vs. control.

Cell binding of [^64^Cu]**3B** was measured using the three prostate cancer cell lines. As shown in Figure 3A, cell binding of [^64^Cu]**3B** increased in the order of PC3, 22Rv1, and LNCaP. The binding also increased in a time-dependent manner from 1–24 h (LNCaP: 1.64–3.33% ID/mg; 22Rv1: 1.17–2.46% ID/mg; PC3: 0.70–1.92% ID/mg) (Figure 3A). A blocking study at 6 h demonstrated that binding of [^64^Cu]**3B** to LNCaP cells was reduced by 45.7% (*** *p*) and that to 22Rv1 cells was reduced by 50.5% (*** *p*) in the presence of **3B** (Figure 3B), suggesting relatively higher specific binding of the radioligand to 22Rv1 than to LNCaP cells. Furthermore, binding to PC3 cells was reduced by 23.9%, which was not statistically significant. The results demonstrated that the hepsin level in the 22Rv1 cells was higher than in PC3 but lower than in LNCaP cells (Figure 3). 

### 2.6. MicroPET Imaging

**Figure 4:**
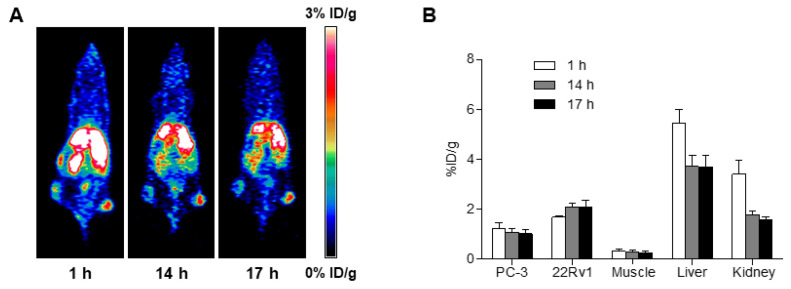
(**A**) MicroPET images of mice inoculated with PC3 (left flank) and 22Rv1 (right flank) cells and (**B**) the ROI analysis of major tissues. The mice were injected with [^64^Cu]**3B** and PET images were acquired at 1, 14, and 17 h after injection.

MicroPET images were acquired using mice in which PC3 and 22Rv1 cells were inoculated in the left and right flanks, respectively (Figure 4A). Region of interest (ROI) analysis demonstrated that the radioligand uptakes in the 22Rv1 tumors (1.67 ± 0.06 at 1 h, 2.07 ± 0.15 at 14 h, and 2.10 ± 0.26 at 17 h) were higher than in the PC3 tumors (1.23 ± 0.22 at 1 h, 1.07 ± 0.14 at 14 h, and 1.02 ± 0.17 at 17 h) at all time points (Figure 4B). The radioligand uptake was high and persistent over time in 22Rv1 tumors, whereas it slowly decreased in PC3 tumors over 17 h after injection. Relatively high uptake was noted in the liver, suggesting that the radioligand was excreted through the hepatobiliary system.

## 3. Discussion

Early and accurate diagnosis of prostate cancer is vital for improving the prognosis of the disease. Hepsin is overexpressed in cancerous prostate but not in normal prostate [7,8,9,10,11]. Moreover, it is located on the cell surface [12], making it an ideal biomarker for PET imaging. Although a few studies have examined the optical imaging of hepsin, PET imaging studies of hepsin specifically have not been conducted. In this study, we designed, synthesized, and investigated a radioligand for PET imaging studies of hepsin. Six ligands were synthesized and evaluated for their binding affinities and selectivity for hepsin (Figure 1). Based on the binding assay results, all six ligands were found to be potent reversible hepsin inhibitors, and **1A** and **1B** had higher binding affinities and selectivity for hepsin compared to the other ligands (Table 1). On the other hand, ligand **3B** was the preferred candidate for the development of a radioligand because of the facile radiolabeling of **11B** with [^64^Cu]CuCl_2_ and its potential use as a theranostic pair, in addition to its potent binding affinity and selectivity for hepsin.

Radiochemical synthesis of [^64^Cu]**3B** which involved the incorporation of ^64^Cu into the DOTA-conjugated compound (**11B**) was straightforward (Scheme 2). However, a small quantity of [^64^Cu]**3A** was also formed, even though the HPLC-purified **11B** was used for radiolabeling. Therefore, [^64^Cu]**3B** was purified by HPLC. The desired HPLC fraction was collected; however, the removal of the HPLC solvents at 80 °C under a gentle stream of N_2_ was time-consuming. Although this procedure could be improved by using solid-phase extraction cartridges, the water-solubility of the product would hamper the use of cartridges.

We performed in vitro and in vivo characterization of [^64^Cu]**3B** using prostate cancer cells and a prostate tumor mouse model. LNCaP and PC3 cells were selected as the high and low hepsin expressing cell lines [13,24]. The 22Rv1 cell line was included in this study. The cell binding of [^64^Cu]**3B** was high in LNCaP cells, intermediate in 22Rv1 cells, and lowest in PC3 cells (Figure 3A). Blocking of cell binding using **3B** resulted in 45.7–50.5% inhibition in LNCaP and 22Rv1 cells (*** *p*) at 6 h, but only a 23.9% inhibition in PC3 cells (Figure 3B). The results revealed the hepsin level in the 22Rv1 cells, which was higher than in the PC3 but lower than in the LNCaP cells, and the specificity of [^64^Cu]**3B** for hepsin. 

Early detection of tumors is clinically important [13]. Therefore, we conducted PET imaging studies on small tumors in mice that were implanted with PC3 cells on the left flank and 22Rv1 cells on the right flank (Figure 4A). The 22Rv1 cells lines were selected for the mouse tumor model owing to their fast growth rate and high self-blocking levels as compared to LNCaP cell lines. (Figure 3). In contrast, the PC3 was selected as the low hepsin-expressing cell lines. Based on the ROI analysis of tumor PET images, the radioligand uptake at 17 h after injection was two-fold higher in 22Rv1 tumors than that in PC3 tumors (Figure 4B). Moreover, the radioligand uptake in 22Rv1 tumors increased over time, whereas the uptake in PC3 tumors decreased, suggesting that tumor uptake was due to the specific binding of [^64^Cu]**3B** to hepsin. In this study, the radioligand was evaluated in mice implanted with two cell lines displaying varying levels of hepsin (Figure 3). However, in vivo blocking studies may be needed to further support the hepsin specificity of [^64^Cu]**3B** in vivo.

An in vitro serum stability study showed that the [^64^Cu]**3B** was slowly epimerized to the corresponding A isomer when incubated with FBS at 37 °C (Figure 2). Although the observed in vitro serum stability of the radioligands may not always reflect their in vivo stability, this in vitro study is widely used for predicting in vivo stability of radioligands. In this study, PET images were acquired at 1, 14, and 17 h after radioligand injection. Based on the in vitro serum stability study findings, the PET images obtained at 17 h could be attributed to the radioligand that was comprised of [^64^Cu]**3B** (higher than 70.3%) and [^64^Cu]**3A** (less than 23.9%) (Figure 2). The PET images acquired within a few hours using a radioligand with a shorter half-life will reveal an uptake only by [^64^Cu]**3B** in vivo. Although further studies are warranted to investigate the in vivo epimerization of the [^64^Cu]**3B** to the [^64^Cu]**3A**, the [^64^Cu]**3B** was mostly detected during the entire duration of the in vitro stability study. Furthermore, both **3A** and **3B** were found to be potent hepsin inhibitors (Table 1). Alternatively, a diastereomeric mixture of [^64^Cu]**3A** and [^64^Cu]**3B** can be used for hepsin imaging. 

Most of the reported hepsin inhibitors exhibit high binding affinities but relatively low selectivity for hepsin over matriptase and other proteases [15,23,25,26,27,28,29,30]. The ligands developed in this study exhibited superior binding affinities with a moderate selectivity for hepsin (Table 1). To this end, the development of radioligands with both higher binding affinities and selectivity for hepsin is required, thus allowing for their improved uptake in hepsin-expressing prostate tumors.

## 4. Materials and Methods

### 4.1. General Information 

Chemicals were purchased from Merck (Darmstadt, Germany), Tokyo Chemical Industry (Tokyo, Japan), and CheMatech (Dijon, France). ^1^H and ^19^F NMR spectra were obtained using Bruker Avance III 600 (600 MHz) and 500 (500 MHz) spectrometers (Rheinstetten, Germany). Chemical shifts (δ) are reported as the ppm downfield of the internal standard tetramethylsilane. Electrospray ionization (ESI) mass spectra were obtained using a compact ESI-Q-TOF MS/MS system (Bruker, Rheinstetten, Germany). All buffers used for the synthesis of non-radioactive ligands (**2** and **3**) were pretreated with Chelex 100 resin to ensure that they are metal-free. The aqueous HPLC eluents used for the purification of the DOTA-conjugated compound (**11**) and non-radioactive ligands (**2** and **3**) were also pretreated with Chelex 100 resin. Purification and analyses of the ligands were performed by HPLC (Thermo Fisher Scientific, Waltham, MA, USA) equipped with a semi-preparative column (YMC-Pack C18, 5 µ, 10 × 250 mm) and an analytical column (YMC-Pack C18, 5 µ, 4.6 × 250 mm), respectively. The eluent was monitored using a UV (230 nm) detector.

### 4.2. Chemical Synthesis

#### 4.2.1. (*S*)-*tert*-Butyl 4-Methyl-2-(pent-4-ynamido)pentanoate (**4**)

1-Hydroxybenzotriazole (HOBt) (1.65g, 12.23 mmol) and *N*-ethyl-*N’*-(3-dimethylaminopropyl)carbodiimide (EDC)·HCl (2.9 g, 12.13 mmol) were slowly added to a stirred solution of 4-pentynoic acid (1 g, 10.19 mmol) in CH_2_Cl_2_ (100 mL) at room temperature. After 20 min of stirring, l-leucine *tert*-butyl ester∙HCl (2.7 g, 12.23 mmol) and Et_3_N (2.13 mL, 15.29 mmol) were added to the solution. The reaction mixture was stirred for 3 h, after which, it was quenched with a saturated NaHCO_3_ solution (aq.) and extracted with ethyl acetate. The organic layer was then washed with brine, dried over MgSO_4_, filtered, and concentrated in vacuo. The crude residue was purified using flash column chromatography (3:1 hexane:ethyl acetate) to yield compound **4** (2.61 g, 96%) as a colorless oil. ^1^H NMR (500 MHz, CDCl_3_) *δ* 5.97 (d, *J* = 8.4 Hz, 1H), 4.57–4.51 (m, 1H), 2.56–2.50 (m, 2H), 2.47–2.40 (m, 2H), 1.99 (t, *J* = 2.5 Hz, 1H), 1.72–1.66 (m, 1H), 1.65–1.58 (m, 1H), 1.53–1.49 (m, 1H), 1.47 (s, 9H), 0.95 (d, *J* = 2.5 Hz, 3H), 0.94 (d, *J* = 2.5 Hz, 3H). HRMS (ESI) *m*/*z* [M+H]^+^ calcd for C_15_H_26_NO_3_, 268.1913; found, 268.1907.

#### 4.2.2. (*S*)-4-Methyl-2-(pent-4-ynamido)pentanoic acid (**5**)

Trifluoroacetic acid (TFA) (10 mL) was slowly added to a stirred solution of compound **4** (2 g, 7.48 mmol) in CH_2_Cl_2_ (50 mL) at 0 °C. The mixture was then stirred at room temperature for 2 h. At the end of the reaction, the solvent and TFA were removed from the reaction mixture. The residue was solidified with diethyl ether, filtered, and dried to yield compound **5** (1.58 g, 100%) as a white solid. ^1^H NMR (500 MHz, CD_3_OD) *δ* 4.45–4.41 (m, 1H), 2.50–2.41 (m, 4H), 2.25–2.22 (m, 1H), 1.78–1.69 (m, 1H), 1.64–1.59 (m, 2H), 0.95 (d, *J* = 6.6 Hz, 3H), 0.92 (d, *J* = 6.5 Hz, 3H). HRMS (ESI) *m*/*z* [M+H]^+^ calcd for C_11_H_18_NO_3_, 212.1287; found, 212.1283.

#### 4.2.3. *N*-((2*S*)-1-(5-(3-((4-Methoxy-2,3,6-trimethylphenyl)sulfonyl)guanidino)-1-oxo-1-(thiazol-2-yl)pentan-2-yl)amino)-4-methyl-1-oxopentan-2-yl)pent-4-ynamide (**6**) 

HOBt (0.38 g, 2.84 mmol) and EDC·HCl (0.54 g, 12.13 mmol) were slowly added to a stirred solution of compound **5** (0.5g, 2.37 mmol) in CH_2_Cl_2_ (50 mL) at 0 °C. After stirring for 20 min, NH_2_–Arg(Mtr)–kt (1.43 g, 2.60mmol) and Et_3_N (0.66 mL, 4.73 mmol) were added at 0 °C. The reaction mixture was stirred at room temperature for 3 h, after which, it was quenched with a saturated NaHCO_3_ solution (aq.) and extracted with ethyl acetate. The organic layer was washed with brine, dried over MgSO_4_, filtered, and concentrated in vacuo. The crude residue was purified by flash column chromatography (1:3 hexane:ethyl acetate) to yield compound **6** (1.37 g, 90%) as a white solid. ^1^H NMR (500 MHz, CD_3_OD) *δ* 8.05 (d, *J* = 3.0 Hz, 1H), 8.00 (d, *J* = 2.9 Hz, 1H), 6.62 (s, 1H), 4.48–4.41 (m, 1H), 3.83 (s, 3H), 3.25–3.15 (m, 2H), 2.63 (s, 3H), 2.57 (s, 3H), 2.49–2.41 (m, 4H), 2.27–2.23 (m, 1H), 2.10 (s, 3H), 2.06–1.95 (m, 1H), 1.73–1.61 (m, 3H), 1.60–1.51 (m, 3H), 1.30–1.26 (m, 1H), 0.97–0.93 (m, 3H), 0.93–0.90 (m, 3H). HRMS (ESI) *m*/*z* [M+H]^+^ calcd for C_30_H_43_N_6_O_6_S_2_, 647.2685; found, 647.2681.

#### 4.2.4. *N*-((2*S*)-1-(5-Guanidino-1-oxo-1-(thiazol-2-yl)pentan-2-yl)amino)-4-methyl-1-oxopentan-2-yl)pent-4-ynamide (**7**) 

Compound **6** (0.65 g, 1.0 mmol) was dissolved in TFA/thioanisole/water (95/2.5/2.5, v/v/v, 20 mL), and the solution was stirred at room temperature for 3 h. After the reaction mixture was cooled to 0 °C, isopropyl ether (60 mL) was added. The resulting white precipitates were filtered, washed with isopropyl ether, and dried to yield compound **7** (0.46 g, 83%) as a white solid. ^1^H NMR (500 MHz, CD_3_OD) δ 8.09 (d, *J* = 3.2 Hz, 1H), 8.03 (d, *J* = 3.1 Hz, 1H), 5.60–5.53 (m, 1H), 4.45–4.38 (m, 1H), 3.27–3.19 (m, 2H), 2.51–2.42 (m, 4H), 2.30–2.25 (m, 1H), 2.17–2.08 (m, 1H), 1.82–1.65 (m, 4H), 1.60–1.52 (m, 2H), 0.98–0.89 (m, 6H). HRMS (ESI) *m*/*z* [M+H]^+^ calcd for C_20_H_31_N_6_O_3_S, 435.2178; found, 435.2173.

#### 4.2.5. (2*S*)-2-(3-(1-(2-(2-(2-Fluoroethoxy)ethoxy)ethyl)-1*H*-1,2,3-triazol-4-yl)propanamido)-*N*-(5-guanidino-1-oxo-1-(thiazol-2-yl)pentan-2-yl)-4-methylpentanamide (**1**)

Compound **7** (25 mg, 0.046 mmol) in water (100 μL) was added to 1-azido-2-(2-(2-fluoroethoxy)ethoxy)ethane (24 mg, 0.14 mmol) in ethanol (100 μL). CuSO_4_·5H_2_O (0.5 M, 0.023 mmol) and sodium ascorbate (0.5 M, 0.046 mmol) were sequentially added to this solution. The reaction mixture was stirred at room temperature for 3 h. The reaction mixture was diluted with water and purified by HPLC using a semi-preparative column eluted with an 80:20 mixture of 0.1% TFA–water and 0.1% TFA–acetonitrile at a flow rate of 3 mL/min. The desired fractions were collected, concentrated in vacuo, and lyophilized to yield **1A** and **1B** as white solids. 

**1A:** ^1^H NMR (600 MHz, CD_3_OD) δ 8.10 (d, *J* = 3.0, 1.7 Hz, 1H), 8.04 (d, *J* = 3.0 Hz, 1H), 7.83 (s, 1H), 5.54 (dd, *J* = 9.9, 3.9 Hz, 1H), 4.56–4.50 (m, 3H), 4.47–4.45 (m, 1H), 4.43–4.35 (m, 1H), 3.90–3.84 (m, 2H), 3.71–3.68 (m, 1H), 3.66–3.63 (m, 1H), 3.63–3.59 (m, 4H), 3.26–3.17 (m, 2H), 3.07–2.96 (m, 2H), 2.62 (t, *J* = 6.7 Hz, 2H), 2.17–2.10 (m, 1H), 1.88–1.80 (m, 1H), 1.77–1.64 (m, 2H), 1.63–1.52 (m, 3H), 0.94 (d, *J* = 6.2 Hz, 3H), 0.89 (d, *J* = 6.0 Hz, 3H). ^19^F NMR (565 MHz, CD_3_OD) δ -76.93. HRMS (ESI) *m*/*z* [M+H]^+^ calcd for C_26_H_43_FN_9_O_5_S, 612.3092; found, 612.3089.

**1B:** ^1^H NMR (500 MHz, CD_3_OD) δ 500 MHz, CD_3_OD) δ 8.10 (d, *J* = 3.1 Hz, 1H), 8.03 (d, *J* = 2.9 Hz, 1H), 7.85 (s, 1H), 5.56 (dd, *J* = 9.6, 3.8 Hz, 1H), 4.57–4.52 (m, 3H), 4.47–4.44 (m, 1H), 4.38 (t, *J* = 7.5 Hz, 1H), 3.88 (t, *J* = 5.1 Hz, 2H), 3.72–3.69 (m, 1H), 3.66–3.60 (m, 5H), 3.24 (q, *J* = 6.6 Hz, 2H), 2.99 (t, *J* = 7.9 Hz, 2H), 2.62 (t, *J* = 7.1 Hz, 2H), 2.17–2.09 (m, 1H), 1.84–1.71 (m, 3H), 1.65–1.52 (m, 3H), 0.95 (d, *J* = 6.4 Hz, 3H), 0.90 (d, *J* = 6.4 Hz, 3H). ^19^F NMR (282 MHz, CD_3_OD) δ -78.98. HRMS (ESI) *m*/*z* [M+H]^+^ calcd for C_26_H_43_FN_9_O_5_S, 612.3092; found, 612.3085.

#### 4.2.6. 1-Amino-*N*-((2*S*)-1-((5-(3-((4-methoxy-2,3,6-trimethylphenyl)sulfonyl)guanidino)-1-oxo-1-(thiazol-2-yl)pentan-2-yl)amino)-4-methyl-1-oxopentan-2-yl)-3,6,9,12-tetraoxapentadecan-15-amide (**9**)

Piperidine (1 mL) was added to a stirred solution of compound **8** (400 mg, 0.39 mmol) in CH_2_Cl_2_ (10 mL). The reaction mixture was stirred at room temperature for 2 h. It was quenched with a saturated NaHCO_3_ solution (aq.) and extracted with ethyl acetate. The organic layer was washed with brine, dried over MgSO_4_, and concentrated in vacuo. The crude residue was purified by flash column chromatography (9:1 dichloromethane:methanol) to yield compound **9** (300 mg, 96%) as a white solid. ^1^H NMR (500 MHz, CDCl_3_) δ 8.06 (dd, J = 19.9, 2.9 Hz, 1H), 7.73 (dd, J = 18.3, 3.0 Hz, 1H), 6.53 (s, 1H), 5.61–5.45 (m, 1H), 4.60 (m, 1H), 4.14–3.48 (m, 19H), 3.26 (s, 3H), 3.10 (m, 1H), 2.70 (s, 3H), 2.62 (s, 3H), 2.12 (s, 3H), 1.74–1.60 (m, 8H), 1.25 (s, 1H), 1.01–0.86 (m, 6H). HRMS (ESI) *m*/*z* [M+H]^+^ calcd for C_36_H_60_N_7_O_10_S_2_, 814.3843; found, 814.3837. 

#### 4.2.7. Tri-*tert*-butyl 2,2’,2’’-(10-((9*S*)-1-imino-9-isobutyl-1-(4-methoxy-2,3,6-trimethyl phenylsulfonamido)-8,11,27-trioxo-6-(thiazole-2-carbonyl)-14,17,20,23-tetraoxa-2,7,10,26- tetraazaoctacosan-28-yl)-1,4,7,10-tetraazacyclododecane-1,4,7-triyl)triacetate (**10**)

DOTA-tris(*tert*-butyl)ester NHS ester∙HPF_6_ (130 mg, 0.16 mmol) was added to a stirred solution of compound **9** (130 mg, 0.16 mmol) and Et_3_N (33 μL, 0.24 mmol) in CH_2_Cl_2_ (5 mL). The reaction mixture was stirred at room temperature for 2 h. It was quenched with a saturated NaHCO_3_ solution (aq.) and extracted with ethyl acetate. The organic layer was washed with brine, dried over MgSO_4_, and concentrated in vacuo. The crude residue was purified by flash column chromatography (10:1 dichloromethane:methanol) to yield compound **10** (162 mg, 74%) as a white solid. ^1^H NMR (600 MHz, CDCl_3_) δ 8.03 (d, 3.0 Hz, 1H), 7.79–7.67 (m, 1H), 6.53–6.47 (m, 1H), 5.62–5.41 (m, 1H), 4.51–4.26 (m, 1H), 3.81 (s, 3H), 3.74 (s, 2H), 3.70–3.52 (m, 14H), 3.51–3.34 (m, 4H), 3.32–3.12 (m, 4H), 3.05–2.86 (m, 4H), 2.68 (d, *J* = 3.1 Hz, 3H), 2.61 (d, *J* = 3.1 Hz, 3H), 2.56–2.48 (m, 2H), 2.37–2.32 (m, 1H), 2.25–2.19 (m, 1H), 2.09 (s, 3H), 2.07–1.98 (m, 2H), 1.93–1.78 (m, 2H), 1.76–1.59 (m, 7H), 1.51–1.39 (m, 27H), 1.35–1.19 (m, 12H), 0.96–0.92 (m, 3H), 0.91–0.88 (m, 3H). HRMS (ESI) *m*/*z* [M+H]^+^ calcd for C_64_H_110_N_11_O_17_S_2_, 1368.7523; found, 1368.7517. 

#### 4.2.8. 2,2′,2″-(10-((9*S*)-1-Amino-1-imino-9-isobutyl-8,11,27-trioxo-6-(thiazole-2-carbonyl)-14,17,20,23-tetraoxa-2,7,10,26-tetraazaoctacosan-28-yl)-1,4,7,10-tetraazacyclododecane-1,4,7-triyl)triacetic acid (**11**)

Compound **10** (123 mg, 90 μmol) was dissolved in 4 mL of TFA/thioanisole/water (95/2.5/2.5, v/v/v). The solution was stirred at room temperature for 6 h, after which, it was cooled to 0 °C, and isopropyl ether (20 mL) was added to it. The white precipitates formed were filtered, washed with isopropyl ether, and then dried (78 mg, 87%). An aliquot of the resulting compound was purified by HPLC using a semi-preparative column eluted with an 82:18 mixture of 0.1% TFA–water and 0.1% TFA–acetonitrile at a flow rate of 3 mL/min. The desired fractions were collected, concentrated in vacuo, and lyophilized to yield **11A** and **11B** as white solids.

**11A:**^1^H NMR (500 MHz, D_2_O) δ 8.13 (d, *J* = 3.1 Hz, 1H), 8.07 (d, *J* = 3.1 Hz, 1H), 5.48–5.42 (m, 1H), 4.41–4.35 (m, 1H), 3.89–3.75 (m, 6H), 3.71–3.59 (m, 16H), 3.54 (s, 3H), 3.47–3.31 (m, 9H), 3.22 (t, *J* = 6.8 Hz, 2H), 3.19–2.94 (m, 8H), 2.62–2.50 (m, 2H), 2.15–2.06 (m, 1H), 1.87–1.76 (m, 1H), 1.74–1.65 (m, 2H), 1.65–1.50 (m, 3H), 0.93 (d, *J* = 6.0 Hz, 3H), 0.88 (d, *J* = 5.8 Hz, 3H). HRMS (ESI) *m/z* [M+2H]^2+^ calcd for C_42_H_75_N_11_O_14_S, 494.7608; found, 494.7730.

**11B:**^1^H NMR (500 MHz, D_2_O) δ 8.13 (d, *J* = 3.0 Hz, 1H), 8.08 (d, *J* = 3.0 Hz, 1H), 5.50–5.44 (m, 1H), 4.40–4.33 (m, 1H), 3.88–3.75 (m, 6H), 3.72–3.60 (m, 16H), 3.55–3.36 (m, 12H), 3.30–3.21 (m, 3H), 3.20–2.98 (m, 7H), 2.65–2.48 (m, 2H), 2.16–2.07 (m, 1H), 1.89–1.79 (m, 1H), 1.76–1.67 (m, 2H), 1.63–1.51 (m, 3H), 0.92 (d, *J* = 6.0 Hz, 3H), 0.87 (d, *J* = 5.9 Hz, 3H). HRMS (ESI) *m/z* [M+H]^+^ calcd for C_42_H_74_N_11_O_14_S, 988.5137; found, 988.5160. *m*/*z* [M+2H]^2+^ calcd for C_42_H_75_N_11_O_14_S, 494.7608; found, 494.7590.

#### 4.2.9. ^nat^Ga-DOTA-conjugated 1-amino-*N*-(1-((5-guanidino-1-oxo-1-(thiazol-2-yl)pentan-2-yl)amino)-4-methyl-1-oxopentan-2-yl)-3,6,9,12-tetraoxapentadecan-15-amide (**2**)

Ga(NO_3_)_3_·xH_2_O (25.1 mg, 98 μmol) in sodium acetate buffer (0.1 M, pH 6) was added to compound **11** (9.9 mg, 10 μmol). The solution was stirred at room temperature for 16 h. The mixture was diluted with water and then purified by HPLC using a semi-preparative column eluted with an 80:20 mixture of 0.1% TFA–water and 0.1% TFA–acetonitrile at a flow rate of 3 mL/min. The desired fractions were collected, concentrated in vacuo, and lyophilized to yield **2A** and **2B** as white solids.

**2A:** HRMS (ESI) *m/z* [M+H]^+^ calcd for C_42_H_71_GaN_11_O_14_S, 1054.4158; found, 1054.4176.

**2B:** HRMS (ESI) *m/z* [M+H]^+^ calcd for C_42_H_71_GaN_11_O_14_S, 1054.4158; found, 1054.4157.

#### 4.2.10. ^nat^Cu-DOTA-conjugated 1-amino-*N*-(1-((5-guanidino-1-oxo-1-(thiazol-2-yl)pentan-2-yl)amino)-4-methyl-1-oxopentan-2-yl)-3,6,9,12-tetraoxapentadecan-15-amide (**3**)

CuCl_2_·2H_2_O (13.6 mg, 79.2 μmol) in water was added to compound **11** (19.6 mg, 19.8 μmol). The blue-colored solution was stirred at room temperature for 16 h. After the mixture was diluted with water, it was purified by HPLC using a semi-preparative column eluted with an 80:20 mixture of 0.1% TFA–water and 0.1% TFA–acetonitrile at a flow rate of 3 mL/min. The desired fractions were collected, concentrated in vacuo, and lyophilized to yield **3A** and **3B** as blue solids.

**3A:** HRMS (ESI) *m/z* [M+H]^+^ calcd for C_42_H_72_CuN_11_O_14_S, 1049.4277; found, 1049.4271.

**3B:** HRMS (ESI) *m/z* [M+H]^+^ calcd for C_42_H_72_CuN_11_O_14_S, 1049.4277; found, 1049.4272.

### 4.3. Radiochemical Synthesis

#### 4.3.1. General Information

[^64^Cu]CuCl_2_ was kindly provided by the Korea Institute of Radiological and Medical Sciences (KIRAMS; Seoul, Korea). All buffers and aqueous HPLC eluents used for radiolabeling were pretreated with Chelex 100 resin. Purification of the product was performed by HPLC (Thermo Fisher Scientific, Waltham, MA, USA) equipped with a semi-preparative column (YMC-Pack C18, 5 µm, 10 × 250 mm). The analysis of the radioligand was performed by HPLC (Agilent Technologies, Santa Clara, CA, USA) using an analytical column (YMC-Pack C18, 5 µm, 4.6 × 250 mm). Eluates were monitored simultaneously using NaI(T1) radioactivity and UV (230 nm) detectors. Radioactivity was measured using a dose calibrator (Biodex Medical Systems, Shirley, NY, USA).

#### 4.3.2. Synthesis of Radioligand [^64^Cu]**3B**

Compound **11B** (20 μg, 0.018 μmol) was dissolved in 150 μL of sodium acetate buffer (0.1 M, pH 6), and [^64^Cu]CuCl_2_ (50 μL) was added to this solution. The reaction mixture was stirred at 80 °C for 20 min. After cooling to room temperature, the reaction mixture was diluted with water. It was then purified by HPLC equipped with a semi-preparative column using an 80:20 mixture of 0.1% TFA–water and 0.1% TFA–acetonitrile at a flow rate of 3 mL/min. The desired product was eluted between 21.8 and 22.6 min and the HPLC solvents were removed under a gentle stream of N_2_ at 80 °C. The radioligand was re-dissolved in saline for in vitro and in vivo studies.

The molar activity was determined by comparing the UV peak area of the desired radioactive peak and those of the various concentrations of non-radioactive ligand using HPLC. This was performed using an analytical column eluted with a 75:25 mixture of water and acetonitrile, both containing 0.1% TFA, at a flow rate of 1 mL/min. The identity of [^64^Cu]**3B** was determined by co-injecting the radioligand and the corresponding non-radioactive ligand into the HPLC system.

### 4.4. In Vitro Binding Assay

Recombinant hepsin (R&D Systems, Minneapolis, MN, USA) was diluted 5.5-fold in TNC buffer (25 mM Tris-HCl, 150 mM NaCl, 5 mM CaCl_2_, 0.01% Triton X-100, pH 8) and incubated at 37 °C. After 24 h, hepsin was diluted in glycerol to 50%. The stock solution (1.2 μM) was stored at −81 °C and diluted in TNC buffer before use.

Ligands (0.03 nM–30 µM) were diluted in DMSO (2% final concentration) and mixed with either activated hepsin or matriptase in 96-well plates. The final assay concentration for both hepsin and matriptase in TNC buffer was 0.3 nM. After an incubation period of 30 min at 37 °C, BOC–QAR–AMC substrate (R&D Systems) was added to hepsin and matriptase assay mixtures. The final substrate concentration was 150 μM in a final reaction volume of 100 μL.

Changes in fluorescence (excitation at 380 nm and emission at 460 nm) were measured at room temperature over 2 h in a BioTek Synergy two-plate reader (Agilent Technologies). Using the GraphPad Prism version 6.02 software (San Diego, CA, USA), a non-linear curve fit was performed to determine the inhibitor IC_50_s from a plot of the mean reaction velocity versus the inhibitor concentration. The IC_50_ values represent the average of three experimental determinations (Figure S1). *K*_i_ values were calculated using the Cheng and Prusoff equation (*K*_i_ = IC_50_/(1 + [S]/*K*_m_)).

### 4.5. In Vitro Serum Stability

[^64^Cu]**3B** (18.5 MBq) dissolved in saline was added to 50% FBS (Gibco, Brooklyn, NY, USA) and incubated at 37 °C with shaking. At the indicated time points (0, 1, 3, 21, and 24 h), an aliquot was taken, treated with the same volume of acetonitrile, and then centrifuged. The supernatants were analyzed by HPLC (Agilent Technologies) equipped with an analytical column using a 75:25 mixture of 0.1% TFA–water and 0.1% TFA–acetonitrile (Figure S2). Eluates were monitored using a NaI(T1) radioactivity detector.

### 4.6. In Vitro Cell Studies

#### 4.6.1. Cell Lines and Culture

The PC3 cell lines were purchased from the Korean Cell Line Bank (Seoul, Korea), and LNCaP cells were purchased from the American Type Culture Collection (ATCC; Manassas, VA, USA). The 22Rv1 cells (ATCC) were kindly provided by Dr. Yong Jin Lee (KIRAMS, Seoul, Korea). All three human prostate cancer cell lines were free of mycoplasma contamination. They were cultured in RPMI-1640 media supplemented with 10% FBS (ATCC), penicillin (100 U/mL), and streptomycin (100 μg/mL) and maintained at 37 °C in a humidified 5% CO_2_ incubator.

#### 4.6.2. Cell Binding

PC3, LNCaP, and 22Rv1 cells were seeded at 1 × 10^6^ cells/well in six-well plates and cultured in RPMI-1640 medium for 24 h. The medium was changed to 5% FBS before the cell binding study.

[^64^Cu]**3B** (185 kBq/5 μL) was added to each well in a final volume of 2 mL. The plates were incubated at 37 °C for 1, 2, 6, and 24 h. The cells were washed three times with PBS and lysed using 0.1 N NaOH. The lysate was counted using a gamma counter. Protein concentrations in the cell lysates were determined using the Bradford method. For the blocking study, cells were incubated with the radioligand in the presence of **3B** (20 μM) at 37 °C for 6 h and then treated as described above. All experiments were performed in triplicate. Data are expressed as mean ± SEM (*n* = 3).

### 4.7. In Vivo Studies

#### 4.7.1. Animals

BALB/c nude mice (male) aged five weeks were used in this study. Mice were provided drinking water and a normal diet ad libitum. They were maintained under a 12 h light–dark cycle and 50% humidity condition at 24 ± 1 °C. MicroPET images were acquired using an Inveon microPET/CT scanner (Siemens Medical Solutions, Knoxville, TN, USA).

#### 4.7.2. MicroPET Imaging

The tumor xenograft model was prepared by subcutaneously inoculating PC3 (7 × 10^6^) and 22Rv1 cells (7 × 10^6^) suspended in 100 μL of a 1:1 mixture of Matrigel and PBS into the left (PC3) and right (22Rv1) flanks of BALB/c nude mice. MicroPET images were acquired for 10 min at 1, 14, and 17 h after intravenous injection of [^64^Cu]**3B** (8.14 ± 0.06 MBq) into mice (*n* = 3) via the tail vein when tumor volumes had reached 119.3 ± 26.5 mm^3^ (PC3) and 147.0 ± 37.0 mm^3^ (22Rv1). The images were reconstructed using the three-dimensional ordered subset expectation maximization and then processed using Siemens Inveon Research Workplace 4.2. ROIs were drawn over the tumors in the left and right flanks and other major tissues, and the average signal levels in the ROIs were measured. Data are expressed as mean ± SD (*n* = 3).

### 4.8. Statistical Analysis

Data were analyzed using an unpaired, two-tailed Student’s *t*-test using GraphPad Prism version 7.0 software, and differences at the 95% confidence level (*P* < 0.05) were considered significant.

## 5. Conclusions

In this study, we developed a novel PET radioligand for hepsin imaging in prostate tumor. In vitro cell binding study and in vivo characterization of the radioligand in mice, implanted with two prostate cancer cell lines displaying varying levels of hepsin, showed that [^64^Cu]**3B** exhibits desirable characteristics for PET imaging of hepsin. This is the first PET radioligand for hepsin imaging, and our study can be used as a foundation to further develop and refine radioligands for hepsin imaging.

## Figures and Tables

**Table 1:** *K*_i_ (nM) of ligands for hepsin and matriptase.

Ligand	*K*_i_ (nM)		Matriptase/ Hepsin
	Hepsin	Matriptase	
Ac–Leu–Arg–kt	7.8 ± 2.8	56.5 ± 2.7	7.2
**1A**	0.9 ± 0.1	54.5 ± 4.9	60.6
**1B**	0.5 ± 0.1	20.0 ± 0.6	40.0
**2A**	14.3 ± 1.2	166.5 ± 8.2	11.6
**2B**	5.7 ± 0.2	68.2 ± 2.4	12.0
**3A**	15.0 ± 0.6	248.9 ± 12.3	16.6
**3B**	5.1 ± 0.3	119.8 ± 3.5	23.5

## Data Availability

Data is contained within the article and supplementary material.

## Supplementary Materials

The following supporting information can be downloaded at: https://www.mdpi.com/article/10.3390/ph15091109/s1, Analysis data of ligands (NMR) and radioligand (HPLC); Figure S1: IC_50_ curves; Figure S2: In vitro serum stability of [^64^Cu]**3B**.
