# Supplementary material for: A High-Affinity 64Cu-Labeled Ligand for PET Imaging of Hepsin: Design, Synthesis, and Characterization
Source: Pharmaceuticals (Basel). 2022 Sep 5;15(9):1109. doi: 10.3390/ph15091109 (PMC9503212; doi:10.3390/ph15091109)
Supplement: Supplementary file 1 [file pharmaceuticals-15-01109-s001.zip › pharmaceuticals-1874489-supplementary.pdf]

Supporting Information

A High-Affinity ^{64}Cu-Labeled Ligand for PET Imaging of Hepsin: Design, Synthesis, and Characterization

Ji-Hun Park,[1] Xuran Zhang,[1] Hyunsoo Ha,[2] Jung Young Kim,[3] Joon Young Choi,[1] Kyung-Han Lee,[1,4] Youngjoo Byun,[2] and Yearn Seong Choe[1,4,*]

1 Department of Nuclear Medicine, Samsung Medical Center, Sungkyunkwan University School of Medicine, Seoul 06351, Korea

2 College of Pharmacy, Korea University, Sejong 30019, Korea

3 Division of Applied RI, Korea Institute of Radiological and Medical Sciences, Seoul 01812, Korea

4 Department of Health Sciences and Technology, SAIHST, Sungkyunkwan University, Seoul 06355, Korea

Contents:

^{1}H and ^{19}F NMR spectra of **1A** and **1B** 2
^{1}H NMR spectra of **11A** and **11B** 4
HPLC chromatograms of [^{64}Cu]**3B** 5
Figure S1. IC_{50} curves 6
Figure S2. In vitro serum stability of [^{64}Cu]**3B** 7

^{1}H and ^{19}F NMR spectra of 1A and 1B

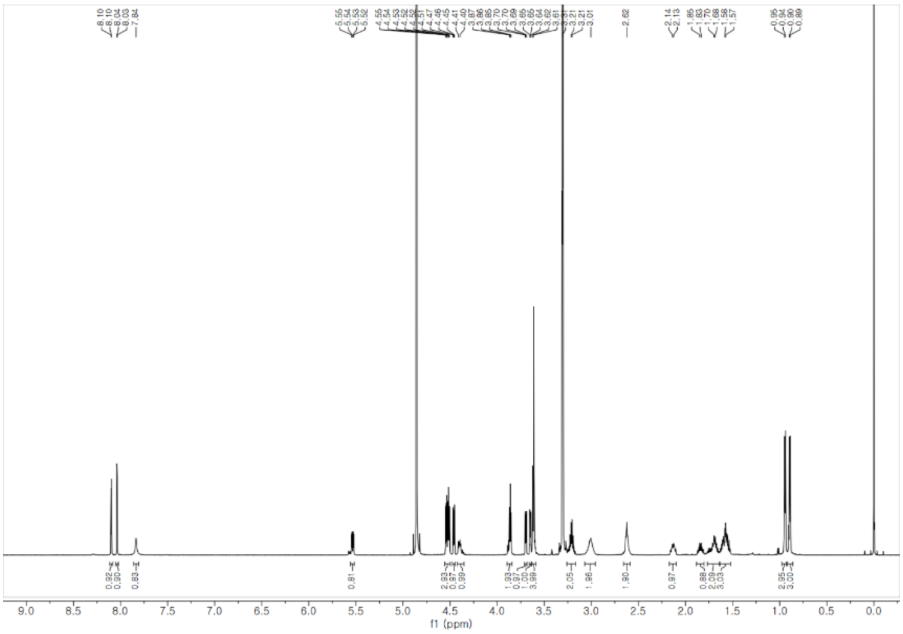

^{1}H NMR spectrum of ligand **1A**

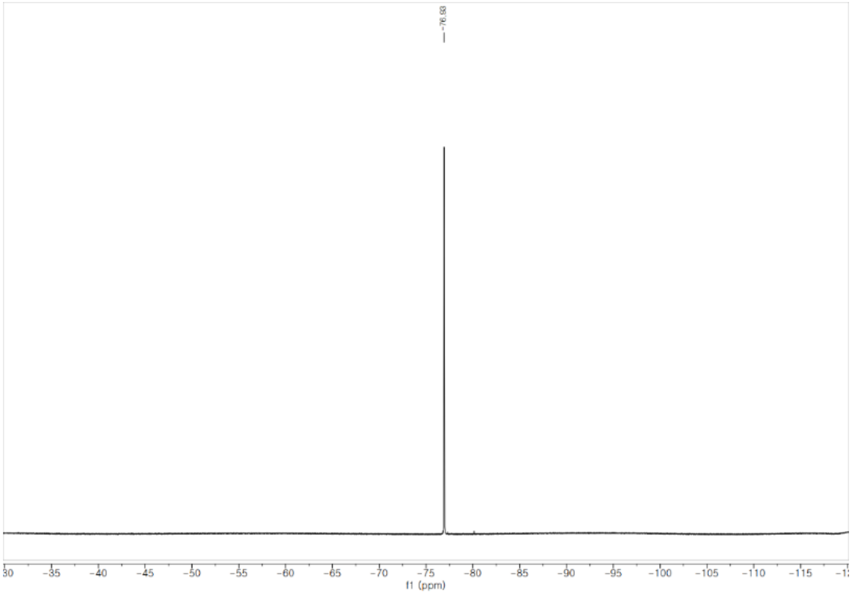

^{19}F NMR spectrum of ligand **1A**

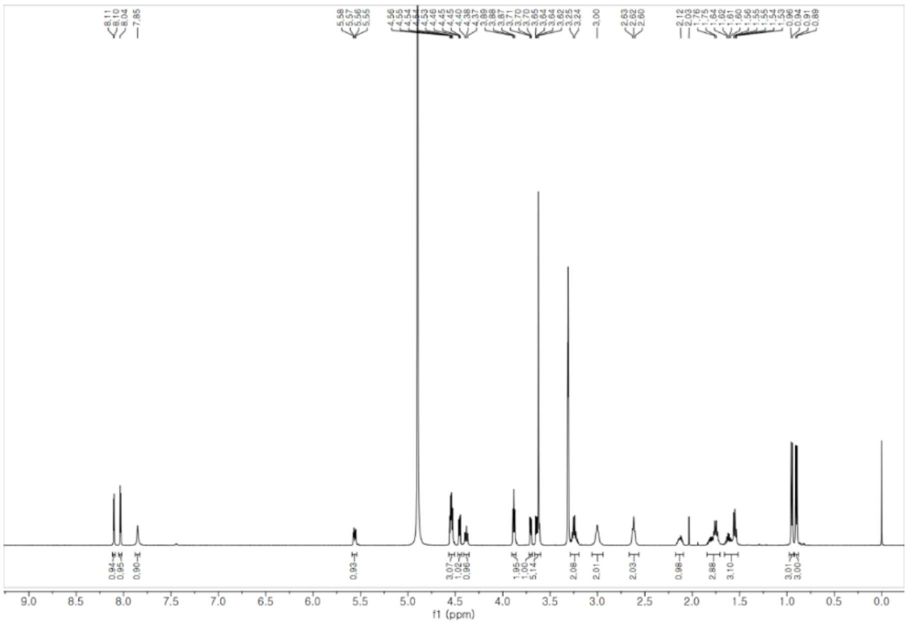

^{1}H NMR spectrum of ligand **1B**

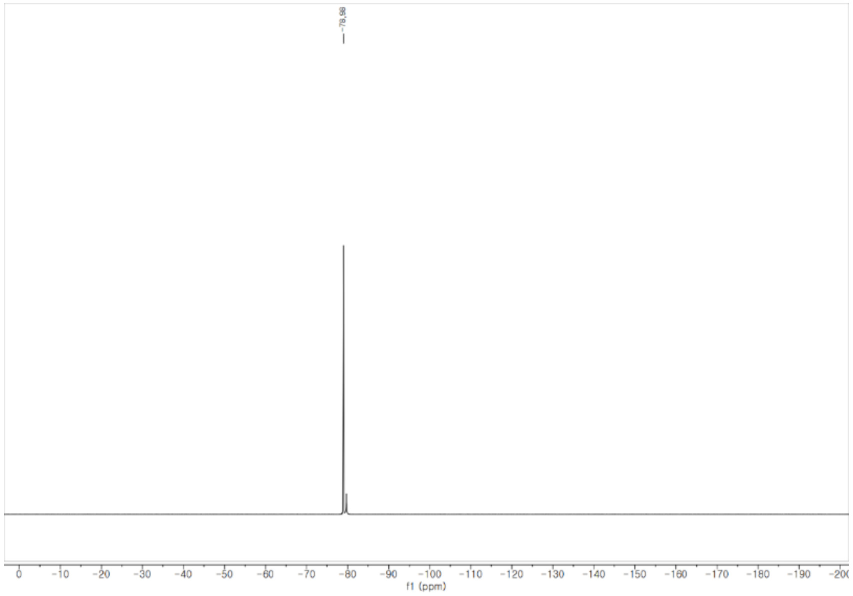

^{19}F NMR spectrum of ligand **1B**

^{1}H NMR spectra of **11A** and **11B**

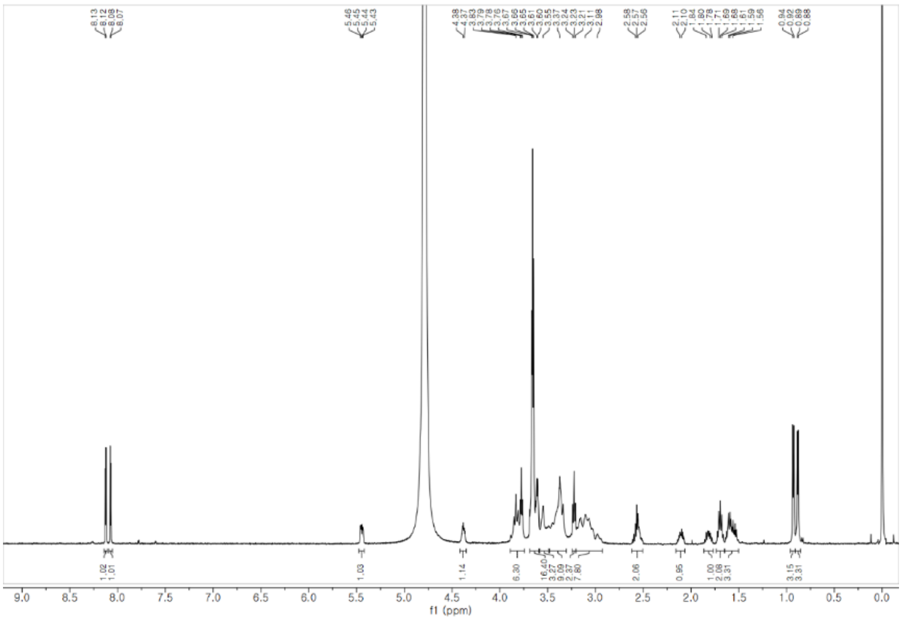

^{1}H NMR spectrum of **11A**

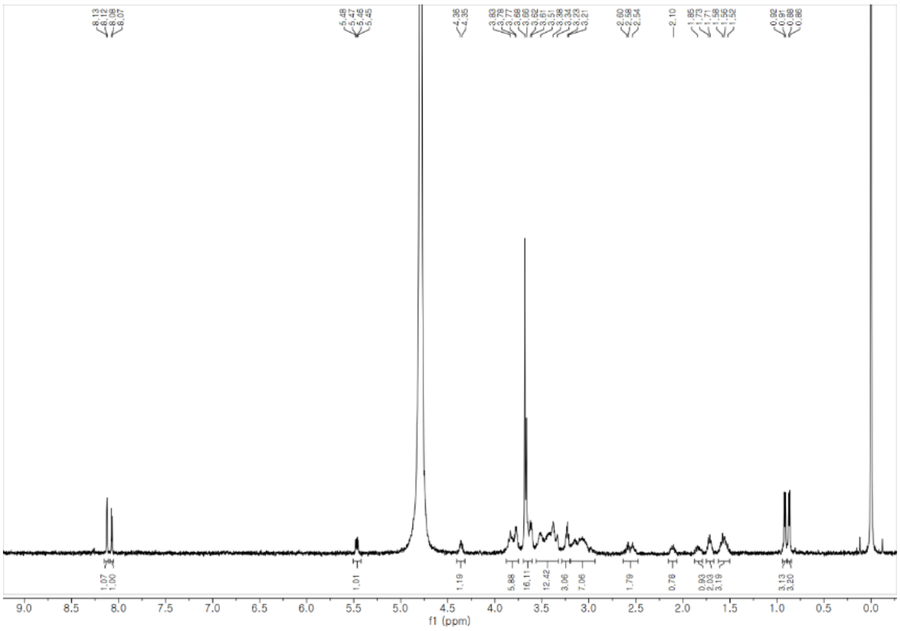

^{1}H NMR spectrum of **11B**

HPLC chromatograms of [^{64}Cu]3B

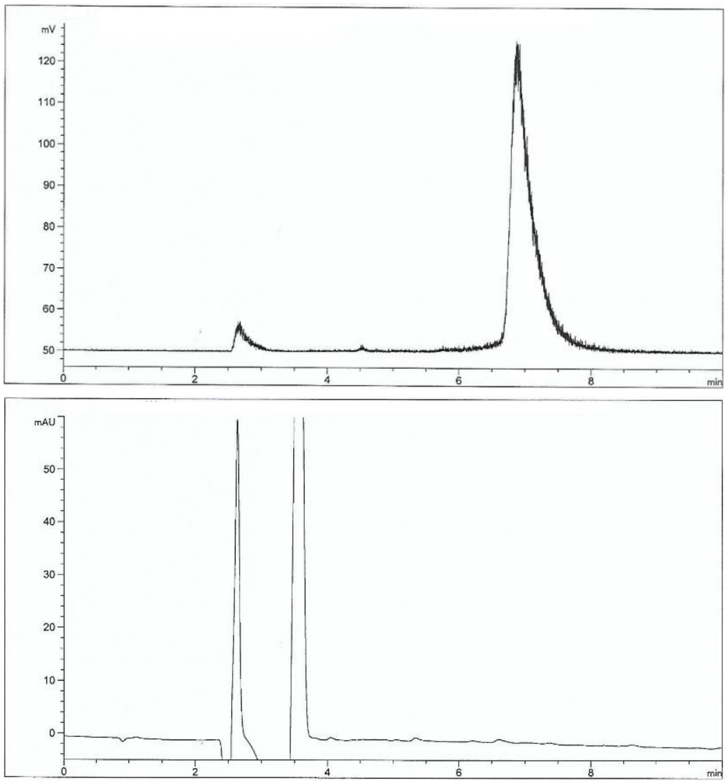

HPLC column: YMC-Pack C18, 4.6 × 250 mm, 5 µm
HPLC solvents: 75:25 water (0.1%TFA) – acetonitrile (0.1%TFA); flow rate: 1 mL/min
Detection: Radioactivity detector (top) and UV detector (230 nm) (bottom)

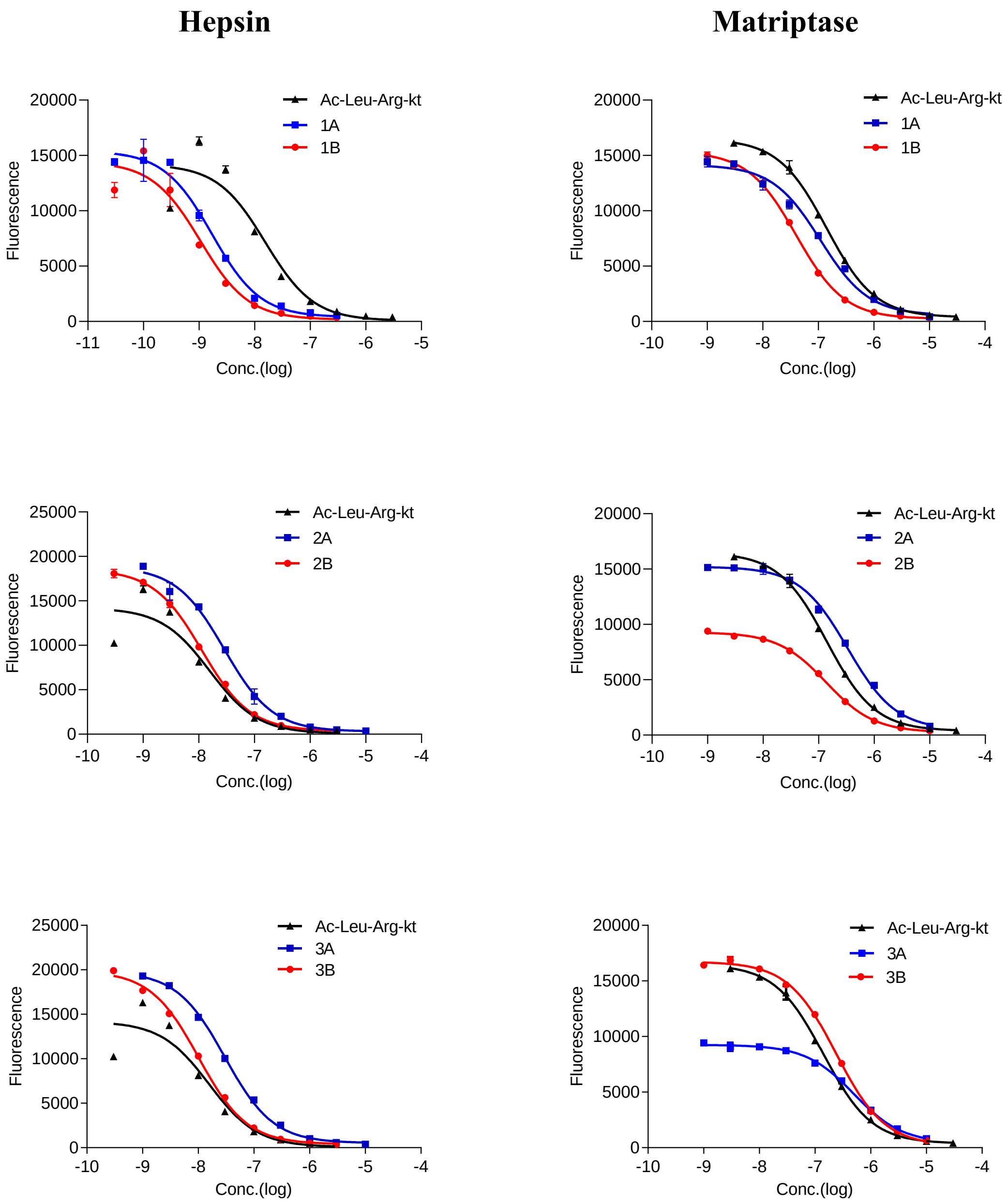

Figure S1. IC$_{50}$ curves of all six ligands for hepsin (left) and matriptase (right).

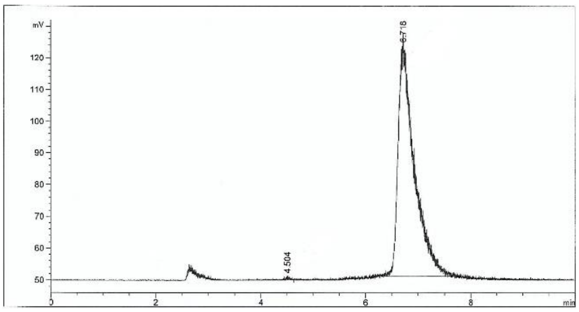

0 h

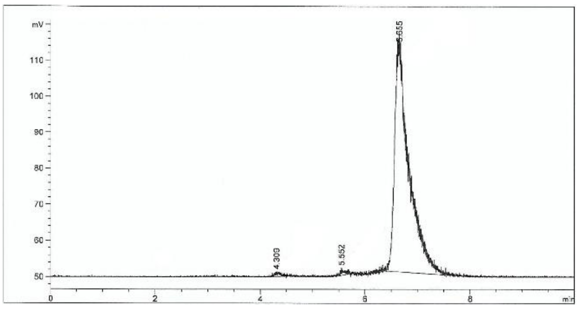

1 h

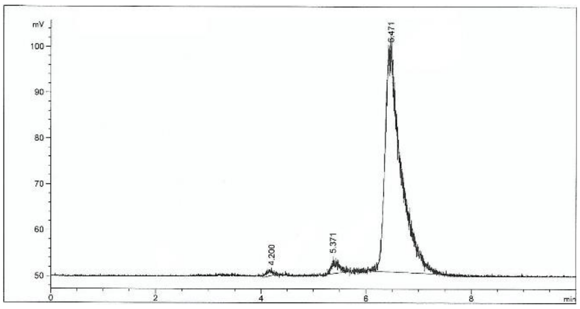

3 h

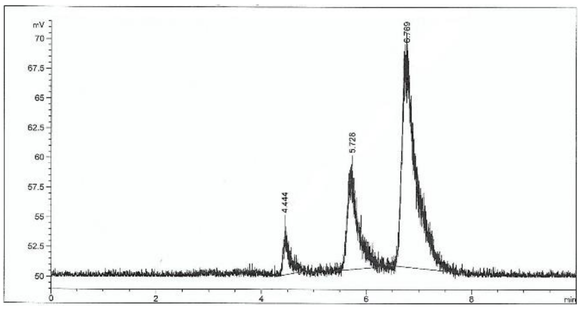

21 h

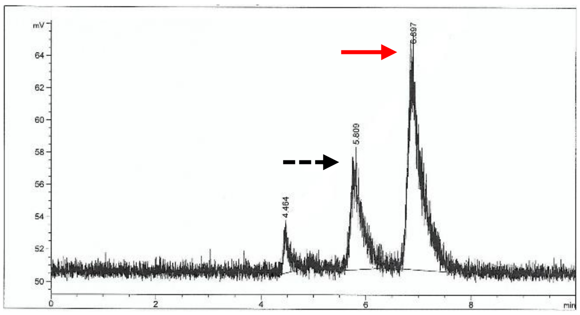

24 h

[^{64}Cu]**3B** (red solid arrow); [^{64}Cu]**3A** (black dotted arrow)

HPLC column: YMC-Pack C18, 4.6 × 250 mm, 5 μm
HPLC solvents: 75:25 water (0.1%TFA) ─ acetonitrile (0.1%TFA)
Flow rate: 1 mL/min
Detection: Radioactivity detector

Figure S2. In vitro serum stability of [^{64}Cu]**3B**.
